# Digital Cushion Fatty Acid Composition and Lipid Metabolism Gene Network Expression in Holstein Dairy Cows Fed a High-Energy Diet

**DOI:** 10.1371/journal.pone.0159536

**Published:** 2016-07-21

**Authors:** Zeeshan Muhammad Iqbal, Haji Akbar, Afshin Hosseini, Elena Bichi Ruspoli Forteguerri, Johan S. Osorio, Juan J. Loor

**Affiliations:** 1 Department of Animal Sciences and Division of Nutritional Sciences, University of Illinois, Urbana, Illinois, 61801, United States of America; 2 Department of Biosciences COMSATS Institute of Information Technology, Islamabad, 44000, Pakistan; 3 Veterinary Clinical Medicine, University of Illinois, Urbana, Illinois, 61801, United States of America; 4 Department of Dairy Science, South Dakota State University, Brookings, South Dakota, 57007, United States of America; Laval University, CANADA

## Abstract

The hoof digital cushion is a complex structure composed of adipose tissue beneath the distal phalanx, i.e. axial, middle and abaxial fat pad. The major role of these fat depots is dampening compression of the corium underneath the cushion. The study aimed to determine expression of target genes and fatty acid profiles in the hoof of non-pregnant dry Holstein cows fed low (CON) or high-energy (OVE) diets. The middle fat pad of the hoof digital cushion was collected soon after slaughter. Despite the lack of effect on expression of the transcription regulators *SREBF1* and *PPARG*, the expression of the lipogenic enzymes *ACACA*, *FASN*, *SCD*, and *DGAT2* was upregulated with OVE. Along with the upregulation of *G6PD* and *IDH1*, important for NADPH synthesis during lipogenesis, and the basal glucose transporter *SLC2A1*, these data indicated a pro-lipogenic response in the digital cushion with OVE. The expression of the lipid droplet-associated protein *PLIN2* was upregulated while expression of lipolytic enzymes (*ATGL*, *ABDH5*, and *LIPE*) only tended to be upregulated with OVE. Therefore, OVE induced lipogenesis, lipid droplet formation, and lipolysis, albeit to different extents. Although concentration of monounsaturated fatty acids (MUFA) did not differ, among the polyunsaturated fatty acids (PUFA), the concentration of 20:5n3 was lower with OVE. Among the saturated fatty acids, 20:0 concentration was greater with OVE. Although data indicated that the hoof digital cushion metabolic transcriptome is responsive to higher-energy diets, this did not translate into marked differences in the fatty acid composition. The decrease in concentration of PUFA, which could contribute to synthesis of inflammatory molecules, in OVE-fed cows indicated that feeding higher-energy diets might be detrimental for the mediation of inflammation in digital cushion. This effect could be further exacerbated by physiologic and endocrine changes during the peripartal period that favor inflammation.

## Introduction

Lameness is one of the most important welfare issues of high-producing dairy cows in North America [1]. The latter has been attributed to management and environmental factors [2] as well as physiologic adaptations such as postpartal negative energy balance, which can lead cows to excessive loss of body condition (BCS) [3]. Postpartal negative energy balance can be exacerbated by prepartal high-energy diets, where overconditioned cows entering a new lactation mobilize greater amounts of fatty acids (FA) from fat depots such as subcutaneous and mesenteric. The excessive FA mobilization may also affect other lipid-rich tissues such as the digital cushion (i.e., the complex structure located underneath the distal phalanx of the claw) by reducing its thickness and consequently compromising its shock-absorbing capacity [4]. A recent study demonstrated a steady decrease in digital cushion mass from calving to 120 d postpartum, at which point its thickness reached the lowest point during lactation [3]. A lower BCS at calving has been associated with reduced thickness of the digital cushion and, in turn, this condition can lead to development of hoof-related diseases such as sole ulcers and white line disease [3].

Differences between perirenal and subcutaneous adipose depots and the digital cushion have been reported [4], where digital cushion not only had lower lipid content but also higher concentrations of monounsaturated (MUFA) than saturated (SFA) FA with the opposite reported for perirenal and mesenteric fat. The authors attributed these effects to the fundamental differences between tissues, i.e. digital cushion vs. adipose tissues, where the lower lipid content in digital cushion denotes a more protective “cushioning” function for the very sensitive corium to the pressure exerted between the horn and the bone. Those functions contrast the well-established energy storage function of adipose tissues [4]. Despite the fact that the digital cushion thickness decreases through early lactation [3], the effect of dietary energy concentration on digital cushion thickness and risk for lameness remains unknown.

Although there is a considerable amount of data at a transcriptomic level regarding the effects of plane of dietary energy [5] on physiologic adaptations during the peripartal period [6] in subcutaneous adipose tissue, there is a lack of transcriptomic data for the digital cushion. Important transcription factors such as peroxisome proliferator-activated receptors (PPAR) and sterol regulatory element-binding transcription protein 1 (SREBF1), which can regulate the transcription of several target genes (e.g., *FASN*, *ACACA*, *SCD*) involved in lipid metabolism have been extensively confirmed to play fundamental roles in the lipolytic or lipogenic state of several tissues in ruminants [7,8]. However, the extent of diet effects at the level of the digital cushion and consequently hoof health remains to be uncovered. Therefore, the objective of this study was to investigate the impact of feeding a higher-energy diet on both FA and transcriptomic profiles in the digital cushion of non-pregnant non-lactating dairy cows.

## Materials and Methods

The Institutional Animal Care and Use Committee (IACUC) of the University of Illinois approved all procedures for this study (protocol #12134). Details of the experimental design have been published elsewhere [9]. Briefly, 14 non-lactating non-pregnant Holstein cows with initial BW = 717 ± 39 and initial BCS = 3.31 ± 0.14 were used. All cows were fed a control diet (low-energy, **CON**; NE_L_ = 1.30 Mcal/kg) to meet 100% of National Research Council (NRC) requirements at ad libitum dry matter intake (DMI) for 3 weeks, after which half of the cows were assigned to a higher-energy diet (**OVE**; NE_L_ = 1.60 Mcal/kg) and half of the cows continued on CON for 6 weeks. The OVE diet was fed ad libitum and resulted in cows consuming ~180% of NRC requirements, while CON cows were fed to consume only 100% of NRC. All cows were housed in ventilated indoor pens (10 m × 15 m; photoperiod of 8 hours light and 16 hours dark) with individual electronic transmission gates and transponders (American Calan, Northwood, NH) for access to feed. At the end of the experimental period cows were euthanized by captive bolt at the College of Veterinary Medicine diagnostic facilities (University of Illinois) after an overnight period without feed. Previous research has revealed that the architecture of the axial, middle and abaxial fat pads composing the digital cushion is different [10]. Because the middle fat pad was proposed to be the most-relevant in the context of ulcer development [10], it was chosen for the present study. After exsanguination, the lower right hind-limb was separated with a band saw, and a second cut was done in the lateral claw 2 cm above the coronary band in order to halve it longitudinally and expose the middle fat pad. Subsequently, a sample of tissue was harvested by blunt dissection and immediately frozen in liquid nitrogen.

Procedures for RNA extraction, cDNA synthesis, primer design and validation, and quantitative RT-PCR were exactly as described previously [9]. The quality of RNA samples was assessed using the 2100 Bioanalyzer (Agilent technologies Inc., Santa Clara, CA). The average RNA integrity number (RIN) of samples used was 7.5 ± 0.2. To address aspects of molecular regulation of adipogenesis, lipogenesis, and lipolysis we selected key genes (Table 1) associated with insulin signaling, carbohydrate metabolism, adipogenic and lipogenic transcription regulators, and regulation of lipolysis. Primer sequences are reported in the supplemental material. The normalization of gene expression data was performed using the geometric mean of glyceraldehyde-3-phosphate dehydrogenase (*GAPDH*), beta-actin (*ACTB*) and ribosomal protein S9 (*RPS9*).

**Table 1:** Symbol, full name, and molecular function of the genes studied.

Symbol	Full name	Molecular function of the protein
Insulin signaling		
*INSR*	Insulin receptor	Insulin receptor substrate binding
*IRS1*	Insulin receptor substrate 1	Insulin and inulin-like growth factor binding
*PDPK1*	3-phosphoinositide dependent protein kinase 1	ATP and protein kinase binding
*AKT1*	v-akt murine thymoma viral oncogene homolog 1	Protein serine/threonine kinase activity
*AKT2*	v-akt murine thymoma viral oncogene homolog 1	Protein serine/threonine kinase activity
Adipogenic transcription regulators		
*SREBF1*	Sterol regulatory element binding transcription factor 1	DNA binding; transcription factor activity
*PPARG*	Peroxisome proliferator activated receptor gamma	DNA binding; transcription factor activity
*RXRA*	Retinoid X receptor alpha	DNA binding; transcription factor activity
*CEBPA*	CCAAT/enhancer binding protein alpha	DNA binding; transcription factor activity
*ADIPOQ*	Adiponectin, C1Q and collagen domain containing	Cytokine activity; hormone activity
Lipogenesis		
*ACACA*	Acetyl-CoA carboxylase alpha	Acetyl-CoA carboxylase activity
*FASN*	Fatty acid synthase	Fatty acid biosynthesis
*ACSS2*	Acyl-CoA synthetase short-chain family member 2	Acetate-CoA ligase activity
*G6PD*	Glucose-6-phosphate dehydrogenase	NADP metabolism
*IDH1*	Isocitrate dehydrogenase 1 (NADP+)	NADP metabolism
*SCD*	Stearoyl-CoA desaturase (delta-9-desaturase)	Desaturase activity
*DGAT2*	Diacylglycerol O-acyltransferase 2	Regulation of triglyceride synthesis
Lipolysis		
*ATGL*	Patatin like phospholipase domain containing 2	Triglyceride lipase activity
*ABDH5*	Abhydrolase domain containing 5	Carboxylic acid hydrolase activity
*PLIN1*	Perilipin 1	Lipid binding
*PLIN2*	Perilipin 2	Lipid binding
*LIPE*	Lipase E, hormone sensitive type	Hormone-sensitive lipase activity
*GHR*	Growth hormone receptor	Growth factor binding
Glucose Metabolism		
*LDHA*	Lactate dehydrogenase A	L-lactate dehydrogenase activity
*PCK1*	Phosphoenolpyruvate carboxykinase 1	Glycerol biosynthesis from pyruvate
*SLC2A1*	Solute carrier family 2 member 1	Glucose transport
*SLC2A4*	Solute carrier family 2 member 4	Insulin-induced glucose transport

For determination of FA profile, twenty mg of sample were homogenized in 2 mL of 4:1 methanol and hexane (vol:vol) containing 30 μg of internal standard (TG:17 glyceryl triheptadecanoate) and 100 μg of butylated hydroxytoluene (BHT) solution. The homogenized samples were placed in glass tubes on ice under nitrogen for 20s. Subsequently, samples were placed in a water bath at 100°C and, after addition of 200 μL of acetyl chloride, incubated for 1 h. Samples were then cooled in an ice bath prior to addition of 5 mL K_2_CO_3_ buffer. Samples were vortexed for 1 min and then centrifuged at 2,000 × g at 4°C for 2 min. The upper phase containing FAME was transferred carefully to an auto-sampler glass vial (2 mL). The GC column used was a 15 m × 0.100 mm × 0.10 μm nitroterephthalic-acid-modified polyethylene glycol (PEG) column (DB_FFAP; cat No. 127-32H2; J and W scientific from Agilent Technology, USA). The temperature program included: initial 150°C with a 0.25 min hold; ramp: 35°C/min to 200°C, 8°C/min to 225°C with a 3.2 min hold, followed by 80°C/min to 248°C/min with 14.7 min hold. Carrier gas was H_2_ at a flow rate of 56.4 cm/s and a constant head pressure of 346.6 kPa; FID set at 250°C, air and nitrogen make-up gas flow rate of 400 mL/min and 30 mL/min, respectively, and H_2_ flow 40 mL/min. Split ratio was 200:1 with an auto sampler injection of 2 μL.

### Statistical Analysis

After normalization with the geometric mean of the internal control genes, the real-time quantitative PCR data were log2 transformed before statistical analysis. The gene expression and FA data were analyzed using PROC MIXED of SAS (version 9.4; SAS Institute Inc., Cary, NC). The fixed effect in the model was diet (CON, OVE), and cow was designated as the random effect. Denominator degrees of freedom were computed by SAS with the statement DDFM = kr. Significant treatment effects were declared at *P* ≤ 0.05 and tendencies at *P* ≤ 0.15. For ease of interpretation the gene expression data are the log2-back transformed means that resulted from the statistical analysis.

## Results

There was no diet effect (*P* ≥ 0.22) for most genes related to insulin signaling except for *PDPK1* which was downregulated (*P* = 0.03) in OVE cows (Table 2). Except for a tendency (*P* ≤ 0.13) for greater expression of *PPARG*, *CEBPA*, and *ADIPOQ* in cows fed OVE, the expression of the adipogenic/lipogenic transcription regulators *SREBF1* and *RXRA* was not affected (*P* ≥ 0.51). Despite the lack of effect on *SREBF1*, the expression of *ACACA*, *FASN*, *SCD*, and *DGAT2* was upregulated (*P* ≤ 0.04) but *ACSS2* downregulated (*P* = 0.01) with OVE (Table 2).

**Table 2:** Gene expression in digital cushion of non-lactating and non-pregnant Holstein cows fed to meet estimated energy requirements (CON, n = 7) or to exceed energy requirements (OVE, n = 7).

Gene	CON	OVE	SEM^1^	*P*-value
Insulin signaling				
*INSR*	0.875	0.955	0.16	0.58
*IRS1*	0.955	0.930	0.18	0.89
*PDPK1*	1.193	0.992	0.09	0.03
*AKT1*	1.876	1.720	0.07	0.22
*AKT2*	0.931	1.011	0.14	0.55
Adipogenic transcription regulators				
*SREBF1*	1.016	0.925	0.18	0.61
*PPARG*	0.520	1.038	0.39	0.08
*RXRA*	0.907	1.028	0.19	0.51
*CEBPA*	0.451	0.973	0.51	0.13
*ADIPOQ*	0.294	1.018	0.71	0.09
Lipogenesis				
*ACACA*	0.708	1.137	0.21	0.03
*FASN*	0.465	1.320	0.31	0.002
*ACSS2*	1.259	0.866	0.14	0.01
*G6PD*	0.865	1.117	0.14	0.06
*IDH1*	0.714	1.051	0.22	0.07
*SCD*	0.268	1.515	0.61	0.01
*DGAT2*	0.296	1.209	0.67	0.04
Lipolysis				
*ATGL*	0.631	1.036	0.31	0.11
*ABDH5*	0.838	1.034	0.12	0.09
*PLIN1*	0.833	0.915	0.25	0.70
*PLIN2*	0.653	1.091	0.22	0.02
*LIPE*	0.363	0.928	0.57	0.10
*GHR*	0.594	1.043	0.27	0.05
Glucose Metabolism				
*LDHA*	0.928	0.980	0.16	0.74
*PCK1*	0.410	0.857	0.59	0.21
*SLC2A1*	0.380	0.625	0.28	0.08
*SLC2A4*	0.650	0.929	0.30	0.23

^1^SEM = Largest standard error of the mean (log-2 scale).

The expression of *G6PD* and *IDHI*, both important for generation of NADPH during lipogenesis, tended (*P* ≤ 0.07) to be greater with OVE (Table 2). However, the expression of *LDHA*, *PCK1*, and *SLC2A4*, which are associated with glucose metabolism and glyceroneogenesis, was not affected (*P* ≥ 0.21) by feeding OVE. Expression of the basal glucose transporter *SLC2A1* tended (*P* = 0.08) to be upregulated with OVE (Table 2). Among genes encoding lipolytic proteins, the expression of the lipid droplet-associated protein *PLIN2* was upregulated (*P* ≤ 0.05) but *PLIN1* did not change (*P* = 0.70) in cows fed OVE. Although expression of *GHR* was upregulated (*P* = 0.05) by OVE, expression of *ATGL*, *ABDH5*, and *LIPE* only tended (*P* ≤ 0.11) to be upregulated (Table 2).

The concentration of MUFA of digital cushion tissue was not affected (*P* ≥ 0.16) by OVE (Table 3). However, eicosenoic acid (20:1n9) tended (*P* = 0.12) to be greater with OVE. Among the PUFA, eicosapentaenoic acid (20:5n3) was the only one affected by feeding OVE, which led to a lower (*P* = 0.04) concentration. Similar to 20:5n3, the concentration of dihomo-gamma-linolenic acid (20:3n6), arachidonic acid (20:4n6), docosadienoic acid (22:2n6), docosapentaenoic acid (22:5n3), and docosahexaenoic acid (22:6n3) tended (*P* ≤ 0.15) to be lower with OVE (Table 3). In contrast to all the above PUFA, eicosatrienoic acid (20:3n3) was the only FA with a concentration that tended (*P* = 0.10) to increase with OVE. Among the SFA, arachidic acid (20:0) was the only one affected by OVE, which led to greater (*P* = 0.001) concentration (Table 3).

**Table 3:** Fatty acid concentration in digital cushion of non-lactating and non-pregnant Holstein cows fed to meet estimated energy requirements (CON, n = 7) or to exceed energy requirements (OVE, n = 7).

Fatty acid	Common name	CON	OVE	SEM^1^	*P*-value
14:0	Myristic acid	0.54	0.53	0.05	0.92
14:1n5	Myristolenic acid	0.87	0.82	0.09	0.68
16:0	Palmitic acid	10.47	9.96	0.93	0.71
18:0	Stearic acid	2.11	1.49	0.39	0.29
18:1n7	Vaccenic acid	12.48	13.05	0.56	0.48
18:1n9	Oleic acid	59.69	60.69	1.23	0.56
18:2n6	Linoleic acid	2.33	1.87	0.27	0.26
18:3n6	Gamma-linolenic acid	0.07	0.07	0.02	0.97
18:3n3	Alpha-linolenic acid	0.17	0.22	0.04	0.39
20:0	Arachidic acid	0.14	0.37	0.04	< 0.01
20:1n9	Eicosenoic acid	0.35	0.40	0.02	0.12
20:2n6	Eicosadienoic acid	0.039	0.036	0.004	0.61
20:3n6	Dihomo-gamma-linolenic acid	0.15	0.10	0.02	0.08
20:3n3	Eicosatrienoic acid	0.01	0.02	0.004	0.10
20:4n6	Arachidonic acid	0.38	0.15	0.10	0.12
20:5n3	Eicosapentaenoic acid	0.31	0.18	0.04	0.04
22:0	Behenic acid	0.008	0.013	0.004	0.38
22:1n9	Erucic acid	0.004	0.005	0.001	0.81
22:2n6	Docosadienoic acid	0.008	0.006	0.001	0.15
22:4n6	Adrenic acid	0.12	0.07	0.03	0.16
22:5n6	Docosapentaenoic acid	0.007	0.008	0.002	0.84
22:5n3	Docosapentaenoic acid	0.20	0.12	0.03	0.08
22:6n3	Docosahexaenoic acid	0.004	0.001	0.001	0.06
24:0	Lignoceric acid	0.006	0.008	0.002	0.52
24:1n9	Nervonic acid	0.07	0.01	0.03	0.16
Unidentified		9.91	10.34	--	--

^1^SEM = Largest standard error of mean.

## Discussion

During the transition period, dairy cows undergo several metabolic and physiologic adaptations in order to ensure a successful initiation of a new lactation cycle. Among these adaptations, the fat depots (e.g., subcutaneous, mesenteric) change from an adipogenic/lipogenic to a lipolytic state after parturition [11]. This effect is mainly attributed to a state of insulin resistance during the transition period [12]. It has been well-documented that feeding a higher-energy diet (primarily by increasing cereal grains) during the close-up period can induce alterations in the prepartal response of adipose to insulin and consequently alter lipid metabolism pathways, with a net result of enhancing body fat deposition [13,14]. In fact, these alterations on prepartal adipose tissue induced by higher-energy diets involve changes at the transcriptome level [5,15]. However, such transcriptomic adaptations have never been studied in the lipid-rich digital cushion of dairy cows.

Although the cows fed OVE in this study had lower plasma NEFA and greater insulin concentrations [9], this effect did not translate into an upregulation of *INSR*, *IRS1*, *AKT1*, or *AKT2* all of which encode essential kinases related to insulin signaling. Such response is not surprising because the activity of these kinases is not controlled at the transcriptional level (Ji et al., 2012). The tendency for upregulation of the adipogenic transcription regulator *PPARG* and *ADIPOQ* with OVE does support enhanced insulin sensitivity just as it occurs in prepartal subcutaneous adipose tissue (Ji et al., 2012). Further evidence for enhanced insulin sensitivity in the digital cushion are the greater expression of *G6PD*, *IDH1*, and *SLC2A1* also reported in prepartal subcutaneous adipose tissue of cows fed OVE [5].

The adipogenic/lipogenic role of *PPARG*, i.e. promoting maturation of pre-adipocytes, is attributed to the coordinated upregulation of several target genes involved in FA biosynthesis and adipogenesis [16]. The transcription factor *SREBF1* plays a central role in the regulation of de novo FA synthesis in non-ruminants, particularly in the liver [17]. The lack of effect on *SREBF1* with OVE resembles that of Ji et al. [5] and Hosseini et al. [9], where *SREBF1* expression in subcutaneous adipose was not affected by feeding OVE. In contrast, similar to Moisa et al. [18] working with intramuscular fat of steers, the concomitant upregulation of *ACACA*, *FASN*, *SCD*, and *DGAT2* along with *PPARG* due to feeding OVE indicate that insulin sensitivity in digital cushion can be enhanced by high-energy diets just as it occurs in subcutaneous fat. The fact that *SREBF1* seems to play a central role during milk fat depression in dairy cows [19], along with the data from this and previous studies [20], indicates that this transcription regulator might be more important for the control of lipogenesis in the mammary gland instead of adipose tissue.

The results obtained on lipolytic genes in the current experiment were in agreement with those reported for subcutaneous adipose tissue by Ji et al. [5], where feeding a higher-energy diet during the close-up dry period resulted in transient or sustained upregulation of these genes. It is noteworthy that the expression of *PLIN1*, perilipin 1, was unresponsive to OVE in the present study just as reported previously in subcutaneous adipose tissue [5]. It could be possible that regulation of PLIN occurs at a post-transcriptional than transcriptional level [21]. In contrast, the tendency (*P* ≤ 0.11) for a concomitant upregulation of *ATGL* and *ABDH5*, both of which control basal lipolysis [21], with OVE indicated a predisposition towards lipolysis but agrees with data in prepartal subcutaneous adipose tissue in cows fed OVE [5]. Such effect has been reported following PPARγ activation in fully-differentiated adipocytes [22].

Regarding the control of lipolysis in the digital cushion during feeding of higher-energy diets prior to parturition, it is still unclear to what extent *ATGL*, *ABDH5*, and *LIPE* in digital cushion are sensitive to insulin signaling as is the case in subcutaneous adipose tissue [5,23]. A lack of change or an upregulation of *ATGL* and *ABDH5* during the peripartal period in digital cushion would indicate a lipolytic state and, in fact, could serve to provide FA to the hoof/corium tissue. Such mechanism seems biologically relevant because the hoof/corium requires FA for the synthesis of the lipid-rich extracellular matrix, the so-called intercellular cementing substance [24]. This is an essential element for final step in the keratinization process, where mature keratinocytes become glued together, and maintain hoof health [24].

The upregulation of *GHR* with OVE was consistent with previous findings in adipose tissue [25]. Thus, OVE could potentially increase responsiveness of the digital cushion to GH and, in turn, this effect might negatively affect insulin signaling as has been described for adipose tissue [26]. An increase in GH responsiveness could impact negatively overall lipid accumulation in the digital cushion of cows fed higher-energy diets. However, the fact that early lactation is characterized by higher circulating concentrations of GH and the mass of digital cushion also is highest at this time [5] casts some doubts on the anti-adipogenic role of GH at the level of digital cushion. Because the cows fed OVE in this study had lower plasma NEFA [9] it could be speculated that upregulation of *ATGL*, *ABDH5*, and *LIPE* in the digital cushion when circulating insulin is high reflects a process of re-cycling of FA rather than a lack of insulin sensitivity [21].

Regardless of diet, the concentration of MUFA and specifically oleic acid (18:1n9) was the highest among all FA detected, which is consistent with a previous report [4]. It is noteworthy that despite the upregulation of *SCD* in OVE-fed cows, this was not translated into a greater concentration of oleic acid. Therefore, it is possible that any alteration in diet composition with the aim to increase the energy density (i.e., OVE diet) might be partly responsible for this lack of effect. For instance, among the main alterations to the OVE diet from the CON diet was the inclusion of ~5% whole cottonseeds [9]. This oilseed has been associated with reduction in desaturase enzyme activity in adipose tissue of beef cattle [27]. In fact, cottonseeds contain significant amounts of sterculic acid [28], a type of cyclopropenoid FA, which is a well-established inhibitor of SCD activity in mouse [29]. The *SCD* gene was the most abundant gene (>76%; S1 File) in digital cushion regardless of dietary treatment. This is in line with the previously [4] observed greater concentration of MUFA in digital cushion in comparison with adipose tissue. The greater concentration of MUFA likely is one important feature of the shock-absorbing properties of digital cushion in the hoof. Therefore, the need for having greater amounts of *SCD* in this tissue is a priority in order to provide sufficient amounts of oleic acid.

Among the FA measured in this study the concentration of arachidic acid (20:0) was the most-impacted by feeding OVE. In contrast to other 20-carbon unsaturated FA, e.g. arachidonic acid (20:4n6), arachidic acid has received less attention by the scientific community, primarily because 20:4n6 is mainly incorporated into cell membrane lipids (i.e., phospholipids) [30]. However, some have observed an upregulation of *Ppara* by 20:0 in liver of rats fed hyperglycemic diets [31]. If such an effect also occurs with bovine *PPARG*, then it could provide a partial explanation for the concomitant upregulation of *PPARG* and *SCD*. Because it is known that *SCD* can be upregulated by either *PPARG* or *SREBF1* [8,32], based on the current results, it is reasonable to speculate that the upregulation of *SCD* was driven by *PPARG*. Regardless of the potential nutrigenomic effects of 20:0, it is evident that this FA in the digital cushion is responsive to dietary energy level. Further research on the biologic role of this FA within the context of lameness in dairy cows seems warranted.

Alpha-linolenic acid (18:3n3) can be converted into the essential FA eicosapentaenoic acid (EPA; 20:5n3) and docosahexaenoic acid (DHA; 22:6n3) in mammalian tissues [33]. Although alpha-linolenic acid was not affected by OVE, the concentration of EPA and DHA (*P* = 0.06) was lower in OVE-fed cows. Interestingly, EPA, DHA, and arachidonic acid which tended to be lower (*P* = 0.12) with OVE, are precursors of eicosanoids, which in turn are important mediators of inflammation [34]. In fact, metabolites of EPA and DHA such as resolvins, protectins, and maresins are important during the resolution of inflammation [34].

Replacing corn gluten meal and blood meal with fish meal in diets fed to beef cattle as a source of PUFA can considerably increase the concentration of EPA and DHA in muscle [35]. In contrast, it is evident that increasing the energy density of OVE diet by adding ground corn and corn silage (8.5% and 20.9%, respectively), which are sources of oleic (18:1n9) and linoleic acid (18:2n6), in contrast to the CON diet [9], can decrease the concentration of EPA and DHA. Thus, composition of OVE compared with CON could partly explain the decrease in concentration of EPA and DHA in the digital cushion of OVE-fed cows. Taken together, we speculate that overfeeding cows with ground corn and corn silage-based diets could impair the local response and resolution of inflammatory conditions in the hoof. This is particularly important within the context of the periparturient period, where inflammatory stimuli are always present.

Overall, at the molecular and FA composition level, the digital cushion of dairy cows appears highly responsive to plane of dietary energy. Lipogenic genes were consistently upregulated by feeding the higher-energy diet, an effect partly orchestrated by *PPARG* rather than *SREBF1*. This is consistent with an extensive body of research performed on this transcription factor demonstrating that long-chain FA activate PPAR signaling. Arachidic acid was the most responsive FA to the OVE diet, indicating a potentially importance role for this FA in the biology of digital cushion. The decrease in concentration of anti-inflammatory FA (i.e., arachidonic acid, EPA, and DHA) in OVE-fed cows indicates that increasing diet energy density through greater inclusion of cereal grains might be detrimental for the mediation of inflammation in digital cushion. This negative effect could be further exacerbated by physiologic and endocrine changes during the peripartal period.

## Supporting Information

S1 File Table A. Features of primers used for qPCR analysis. Table B. qPCR performance.
(DOCX)
