# Supplementary material for: Digital Cushion Fatty Acid Composition and Lipid Metabolism Gene Network Expression in Holstein Dairy Cows Fed a High-Energy Diet
Source: PLoS One. 2016 Jul 21;11(7):e0159536. doi: 10.1371/journal.pone.0159536 (PMC4956307; doi:10.1371/journal.pone.0159536)
Supplement: S1 File — Table A. Features of primers used for qPCR analysis. Table B. qPCR performance. (DOCX) [file pone.0159536.s001.docx]

**Supplemental File**

**Table A.** Primer sequences (5’ to 3’) for quantitative real time PCR

| Gene | Forward primer | Reverse primer | bp^*^ |
| --- | --- | --- | --- |
| *ABHD5* | CTGCAGATGATGTGGGAAAGC | GACTGCCTGGTTCTCGTGTCA | 100 |
| *ACACA* | CATCTTGTCCGAAACGTCGAT | CCCTTCGAACATACACCTCCA | 101 |
| *ACSS2* | GGTGCAGCTGAAGAAGATCGT | CCCCATGTGGACCTTGTTGT | 101 |
| *ADIPOQ* | GATCCAGGTCTTGTTGGTCCTAA | GAGCGGTATACATAGGCACTTTCTC | 131 |
| *AKT1* | CACGTGCTCTGGACGCTTC | ATGGCGAGGTTCCACTCAAAC | 102 |
| *AKT2* | TCGAAGTCATTCATGGTCACCTT | GACGACCCCATGGACTACAAGT | 110 |
| *ATGL* | CACCAGCATCCAGTTCAACCT | CTGTAGCCCTGTTTGCACATCT | 102 |
| *CEBPA* | GCAAAGCCAAGAAGTCCG | GGCTCAGTTGTTCCACCCGCTT | 102 |
| *DGAT2* | CATGTACACATTCTGCACCGATT | TGACCTCCTGCCACCTTTCT | 100 |
| *FASN* | ACCTCGTGAAGGCTGTGACTCA | TGAGTCGAGGCCAAGGTCTGAA | 92 |
| *G6PD* | CAACCAGCTGTCCAACCACAT | CACCATGAGGTTCTGGACCAT | 100 |
| *GHR* | ATCCAGTCCTAGAGACAAATTCTTCTG | TTAGCCCCATCTGTCCAGTGA | 100 |
| *IDH1* | ATGGCTCTCTTGGCATGATGA | CATTCGGTAGTGACGGGTTACA | 101 |
| *INSR* | CGGAGCTCAGAGATCACGACTAT | AGGTTCACAGTTAAGTGCTCAGATGA | 106 |
| *IRS1* | GGGCTTTTCCTCTTGAGTTTCC | GGAGATCAAGGTTCTTCAAAACGAA | 101 |
| *LDHA* | CAAGAGGTACCACTGCCCAT | CACCTTGGCTAAAGGAACCA | 104 |
| *LIPE* | TCAGTGTCCAAGACAGAGCCAAT | CATGCAGCTTCAGGCTTTTG | 102 |
| *PCK1* | AAGATTGGCATCGAGCTGACA | GTGGAGGCACTTGACGAACTC | 120 |
| *PDPK1* | CAAACTGGTGCCAAGGGTTT | CCTGGACACGAACTCCTTTGA | 102 |
| *PLIN1* | TGGCCACCAGAATAAAGCATG | GCTGACGCTGGACAACAGG | 101 |
| *PLIN2* | TTTATGGCCTCATGCTTTTGC | CTCAGAGCAGACCCCAATTCA | 100 |
| *PPARG* | GAGCCCAAGTTCGAGTTTGC | GGCGGTCTCCACTGAGAATAAT | 100 |
| *RXRA* | CGCTCCTCAGGCAAGCA | TGTCAATCAGGCAGTCCTTGTT | 100 |
| *SCD* | TCCTGTTGTTGTGCTTCATCC | GGCATAACGGAATAAGGTGGC | 101 |
| *SLC2A1* | CCCCCAGAAGGTGATTGAAG | GAACCAATCATGCCTCCCAC | 135 |
| *SLC2A4* | CCTTGGTCCTTGGCGTATTC | TGTAGCTCTGTTCAATCACCTTCTG | 102 |
| *SREBF1* | TGTCCACAAAAGCAAATCGC | TGTCGACCACCTCTGGCTTC | 101 |
| *ACTB* | ACCAACTGGGACGACATCGA | GTCTCGAACATGATCTGGGTCAT |  |
| *RPS9* | CCTCGACCAAGAGCTGAAG | CCTCCAGACCTCACGTTTGTTC |  |
| *GAPDH* | CACTCACTCTTCTACCTTCGATGCT | CCCTGTTGCTGTAGCCAAATTC |  |

*Base pairs

**Table B**. Slope, Coefficient of determination of standard curve (R^2^), efficiency of amplification and median cycle threshold (Ct) of quantitative reverse transcription-PCR

| Gene | Median Ct | Median ∆Ct^1^ | Slope^2^ | (R^2^)^3^ | Efficiency^4^ | mRNA abundance^5^ (%) |
| --- | --- | --- | --- | --- | --- | --- |
| Insulin signaling | | | | | | |
| *INSR* | 25.47 | 4.28 | -3.39 | 0.99 | 1.97 | 0.33 |
| *IRS1* | 27.74 | 6.63 | -3.19 | 0.99 | 2.06 | 0.05 |
| *PDPK1* | 24.09 | 2.95 | -2.98 | 1.00 | 2.17 | 0.61 |
| *AKT1* | 25.64 | 4.59 | -3.33 | 1.00 | 2.00 | 0.25 |
| *AKT2* | 25.16 | 4.04 | -3.39 | 1.00 | 1.97 | 0.39 |
| Adipogenic transcription regulators | | | | | | |
| *SREBF1* | 23.71 | 2.61 | -3.28 | 1.00 | 2.02 | 0.95 |
| *PPARG* | 23.92 | 2.79 | -3.25 | 1.00 | 2.03 | 0.83 |
| *RXRA* | 24.10 | 2.96 | -3.36 | 1.00 | 1.98 | 0.79 |
| *CEBPA* | 24.18 | 3.02 | -3.43 | 1.00 | 1.96 | 0.79 |
| *ADIPOQ* | 21.01 | -0.27 | -3.42 | 0.99 | 1.96 | 7.19 |
| Lipogenesis | | | | | | |
| *ACACA* | 25.48 | 4.29 | -3.25 | 1.00 | 2.03 | 0.29 |
| *FASN* | 21.49 | 0.70 | -3.49 | 0.99 | 1.94 | 3.78 |
| *ACSS2* | 27.23 | 6.04 | -3.29 | 1.00 | 2.02 | 0.09 |
| *G6PD* | 25.09 | 4.04 | -3.41 | 1.00 | 1.96 | 0.39 |
| *IDH1* | 24.10 | 3.08 | -3.29 | 1.00 | 2.01 | 0.69 |
| *SCD* | 17.74 | -3.51 | -3.17 | 1.00 | 2.07 | 76.57 |
| *DGAT2* | 22.98 | 1.95 | -3.27 | 1.00 | 2.02 | 1.52 |
| Lipolysis | | | | | | |
| *ATGL* | 24.47 | 3.35 | -3.37 | 1.00 | 1.98 | 0.61 |
| *ABDH5* | 27.88 | 6.78 | -3.06 | 1.00 | 2.12 | 0.04 |
| *PLIN1* | 26.96 | 5.84 | -3.36 | 1.00 | 1.99 | 0.11 |
| *PLIN2* | 24.90 | 3.73 | -3.36 | 1.00 | 1.98 | 0.47 |
| *LIPE* | 23.18 | 2.15 | -3.24 | 1.00 | 2.03 | 1.30 |
| *GHR* | 23.34 | 2.27 | -3.31 | 0.99 | 2.01 | 1.23 |
| Glucose Metabolism | | | | | | |
| *LDHA* | 24.45 | 3.24 | -3.24 | 1.00 | 2.03 | 0.60 |
| *PCK1* | 28.16 | 7.01 | -3.41 | 1.00 | 1.96 | 0.05 |
| *SLC2A1* | 27.61 | 6.59 | -3.39 | 1.00 | 1.97 | 0.07 |
| *SLC2A4* | 28.61 | 7.74 | -3.19 | 1.00 | 2.06 | 0.02 |

^1^Median ∆Ct is calculated for each gene as [Ct gene – geometrical mean of Ct internal controls].

^2^Slope of standard curve.

^3^Coefficient of determination of the standard curve.

^4^Amplification efficiency [10^(-1/slope)^].

^5^mRNA abundance is percentage of (1/E^∆Ct^) specific gene in the sum of (1/E^∆Ct^) all genes.
